# The urban lives of green sea turtles: Insights into behavior in an industrialized habitat using an animal‐borne camera

**DOI:** 10.1002/ece3.11282

**Published:** 2024-04-25

**Authors:** Cameron M. Mullaney, Jeffrey A. Seminoff, Garrett E. Lemons, Bryant Chesney, Andrew S. Maurer

**Affiliations:** ^1^ University of California San Diego, La Jolla California USA; ^2^ NOAA Southwest Fisheries Science Center La Jolla California USA; ^3^ NOAA West Coast Regional Office Long Beach California USA; ^4^ National Research Council Washington District of Columbia USA

**Keywords:** bay ecosystem, camera, *Chelonia mydas*, life history, reptile, sociality

## Abstract

The cryptic and aquatic life histories of sea turtles have made them a challenging group to directly observe, leaving significant knowledge gaps regarding social behavior and fine‐scale elements of habitat use. Using a custom‐designed animal‐borne camera, we observed previously undocumented behaviors by green turtles (*Chelonia mydas*) at a foraging area in San Diego Bay, a highly urbanized ecosystem in California, USA. We deployed a suction‐cup‐attached pop‐off camera (manufactured by Customized Animal Tracking Solutions) on 11 turtles (mean straight carapace length = 84.0 ± 11.2 cm) for between 1 and 30.8 h. Video recordings, limited to sunlit hours, provided 73 h of total observation time between May 2022 and June 2023. We observed 32 conspecific interactions; we classified 18 as active, entailing clear social behaviors, as compared with 14 passive interactions representing brief, chance encounters. There was no evidence for agonistic interactions. The camera additionally revealed that green turtles consistently use metal structures within urban San Diego Bay. In seven instances, turtles exhibited rubbing behavior against metal structures, and we observed two examples of turtles congregating at these structures. High rates of intraspecific interaction exhibited relatively consistently among individuals provide a compelling case for sociality for green turtles in San Diego Bay, adding to a growing research base updating their historical label of “non‐social.” The frequent use of metal structures by the population, in particular the rubbing of exposed skin, has implications for behavioral adaptations to urban environments. Our study exemplifies the promise of technological advances (e.g., underwater and animal‐borne cameras) for updating natural history paradigms, even for well‐studied populations.

## Graphical abstract

An animal‐borne camera was used to study a population of East Pacific green turtles. Seventy hours of video were recorded, providing insight into the behavioral and social habits of this population.

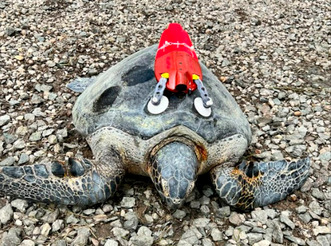

## 1 INTRODUCTION

Sociality reflects the cognitive capacity of an animal population. In both terrestrial and marine systems, mammal sociality has been studied extensively, with much known about aspects of social behaviors ranging from mating systems to communication methods (Aloise King et al., 2013; Thornton & Clutton‐Brock, 2011; Tyack, 1986). However, in other vertebrate groups, sociality has often been assessed only generally, with species being classified as either “social” or “non‐social”; this binary evaluation practice may result in the mischaracterization or dismissal of social tendencies for a variety of species (Hosey et al., 1985; Ruch et al., 2015). The Reptilia are one taxon in particular that has been inherently, and in many cases perhaps wrongly, classified as non‐social (Doody et al., 2009). Indeed, aggregations in time and space of individuals for a variety of Testudines and Squamata have often been attributed to parallel responses to environmental cues rather than social system‐mediated behaviors, a phenomenon most commonly observed in nesting patterns (Doody et al., 2009; Shah et al., 2003). This tendency may be related to historical conceptions about reptile intelligence—or lack thereof—coupled with the cryptic nature of many reptile species, which makes them difficult to observe. Together, these factors have resulted in significant knowledge gaps surrounding sociality within reptile taxa (Doody et al., 2013).

Sea turtles are a group of species whose highly mobile, aquatic, and migratory life histories make them notoriously difficult to directly observe in the wild. The taxon has historically been categorized as non‐social (Gaos et al., 2021; Wilkinson & Huber, 2012). While most opportunities to witness multiple individuals on the same occasion—a key element for studying social behavior—occur at nesting beaches, such occasions are largely due to conspecifics arriving at the beach based on environmental cues, such as temperature and salinity, as opposed to social cues (Barik et al., 2014). However, putative evidence for sociality in sea turtles in aquatic habitats has been reported, albeit infrequently (Campbell, 2010). Communication is one of the clearest indicators of sociality and has been documented in sea turtles by both vocal and chemical means (de Melo et al., 2023; McKenna et al., 2019). Vocalizations by late‐stage developing embryos in buried nests have been recorded in multiple sea turtle species, perhaps reflecting a strategy for synchronized nest emergence (Ferrara et al., 2019; Monteiro et al., 2019), as has been hypothesized for a variety of freshwater turtle species (Baker et al., 2013; Colbert et al., 2010; Ferrara et al., 2019). Limited evidence suggests juvenile sea turtles may vocalize underwater at foraging grounds, though the purpose of these vocalizations remains unclear (Charrier et al., 2022). Potential evidence of social hierarchies among sea turtles has also been suggested, with one study finding larger loggerhead turtles (*Caretta caretta*) maintaining first choice for resting spots over Kemp's ridleys (*Lepidochelys kempii*), greens, and smaller loggerheads in a shared habitat (Lamont et al., 2023). While these are promising examples of sociality in sea turtles, further evidence is needed to determine the prevalence of such behaviors.

Remote tracking techniques such as satellite and acoustic telemetry have become common tools for researching sea turtles and addressing major challenges posed by their cryptic natures (Hardin & Fuentes, 2021; Hays & Hawkes, 2018). Transmitters depict the location of tracked individuals and provide key information about movements and habitat. However, satellite location data are collected only intermittently and can entail significant spatial error, inhibiting assessment of fine‐scale associations between telemetered turtles and their immediate environment. This has resulted in unrefined depictions of interactions between sea turtles and their surroundings. This research gap is shrinking, due in part to the recent increase in animal‐borne camera use in studies of marine megafauna (Moll et al., 2007). Smaller, lighter, and more durable cameras and batteries have led to the discovery of previously unknown behaviors and interactions in many marine species (Fuiman et al., 2002; Williams et al., 2000). Our study utilizes an animal‐borne camera to assess intraspecies and environmental interactions among green turtles assembled at a coastal foraging area in San Diego Bay, California, USA.

San Diego Bay (SDB) is a highly industrialized, temperate bay (5700 ha) in southern California, USA that includes a variety of benthic habitat types, including approximately 17% of the state's total seagrass coverage—dominated by eelgrass, *Zostera marina* (Merkel & Associates, 2020), a key foraging resource (Lemons et al., 2011). Natural hard structure is largely absent in benthic habitats in the bay, though various anthropogenic structures are distributed within benthic habitats. These structures, including sunken vessels and buoy chains, have not been systematically documented. Green turtles are present year‐round at this site, with a most recent estimated abundance of 16–61 in 2009 (Eguchi et al., 2010). Since this estimate, the local population has shown signs of growth (NOAA Unpubl. data), likely owing to decades of successful conservation at the source nesting beaches in Mexico (Delgado‐Trejo & Alvarado‐Diaz, 2012; Early‐Capistrán et al., 2018). The nesting season for the most important source rookery spans from August to January (Delgado‐Trejo & Alvarado‐Diaz, 2012). Previous fine‐scale GPS data have shown significant overlap in the core use areas of individual turtles (Eguchi et al., 2020; MacDonald et al., 2012), though little attention has been given to the possibility of social or environmental interactions taking place at these sites.

## 2 METHODS

**FIGURE 1:**
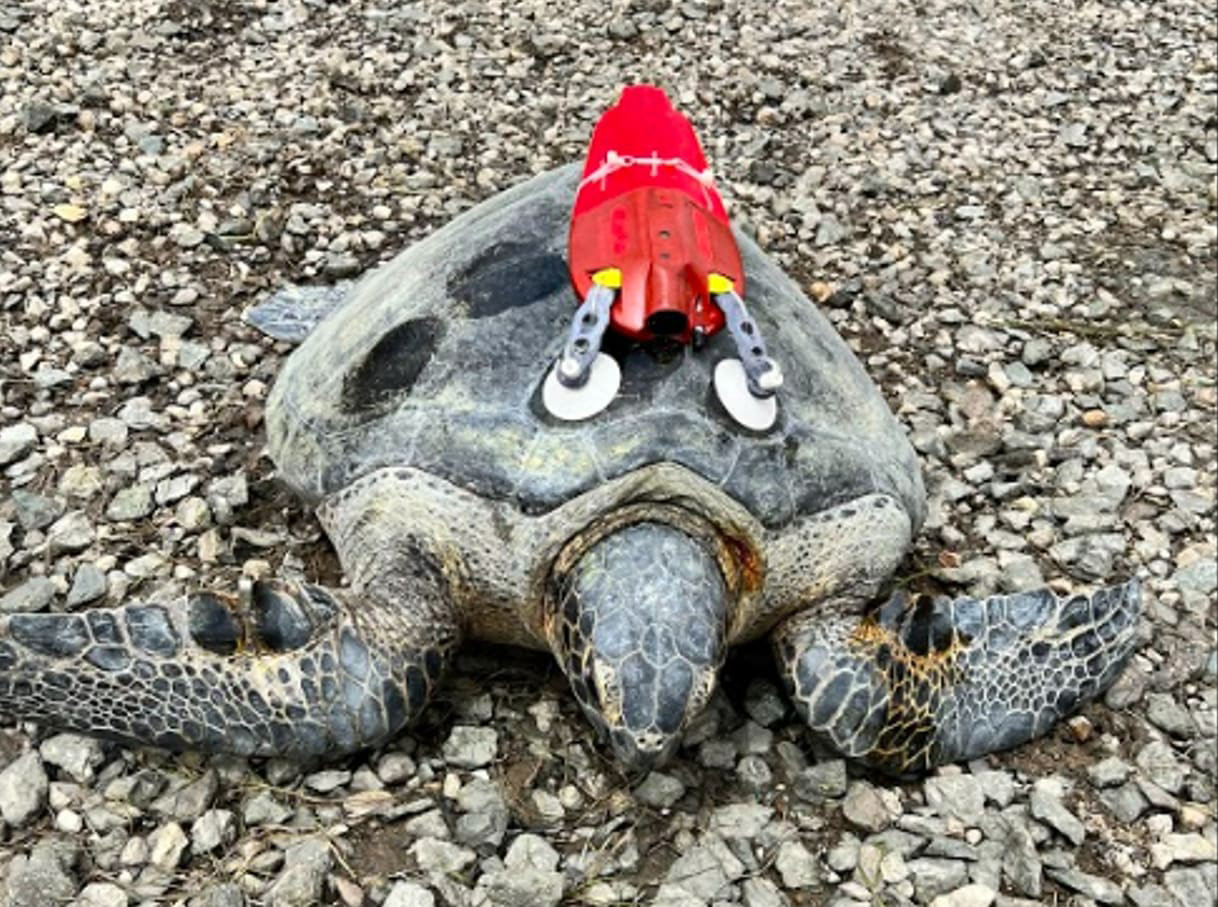
Customized Animal Tracking Solutions (CATS) camera affixed to a green turtle in San Diego Bay, prior to release.

We used a turtle‐borne camera system (Customized Animal Tracking Solutions, Queensland, Australia) to explore the underwater behaviors of green turtles in SDB from May 2022 to June 2023. Deployments took place in southern SDB (32.6134°, −117.0969°). After capture with entanglement nets, turtles were measured and weighed using standard procedures (Table 1, Eguchi et al., 2010). Cameras were attached to turtles with a suction cup apparatus that automatically detached via one of several models of galvanic time‐release mechanisms, with different models used to target deployment durations of 18–36 h, through varying seasonal water temperatures. Camera systems were fitted to the carapace in a forward‐viewing orientation and programmed to record 720p video whenever light availability was sufficient to produce usable video (Figure 1). No sound was recorded. After detachment from the turtle, the positively buoyant camera floated to the surface, activating an Iridium transmitter to provide GPS locations for retrieval. One observer reviewed all footage and demarcated instances of turtle–turtle and anthropogenic structure interactions. Turtle–turtle interactions were categorized as either coincidental (turtles swimming by each other by chance) or active (not by chance; further description below), and active interactions were further categorized as either agonistic or non‐agonistic. To assess whether variation in observed social behavior was explained by body size, we fit two simple linear regressions, one with straight‐carapace length (SCL) as the predictor of interaction rate (interactions per hour) and one with SCL predicting proportion of total video time spent interacting (α = .05). Research was permitted under NOAA Research Permit No. 18238‐03.

**TABLE 1:** Data from 11 deployments on green turtles in San Diego Bay and metadata.

Deployment	Release date (m‐d‐y)	Duration (h)	Video duration (h)	Intraspecies interactions		Sex	SCL (±0.1 cm)	Weight (±0.5 kg)
				Active	Total			
1	5‐26‐2022	6.5	5	0	0	F	78.7	—
2	6‐9‐2022	9	6.5	2	4	U	70.1	46.5
3	7‐6‐2022	21	12	2	5	F	74.3	—
4	8‐16‐2022	30.8	14	5	10	F	86.5	82.0
5	9‐8‐2022	8	5.5	3	4	F	95.3	135.0
6	9‐22‐2022	1	1	0	0	U	70.0	75.0
7	11‐16‐2022	63	14	5	8	F	103.2	148.0
8	3‐3‐2023	1.3	0	NA	NA	F	84.2	69.5
9	5‐2‐2023	28.9	7.8	0	0	F	89.0	101.0
10	5‐9‐2023	20.3	2.5	0	0	M	96.5	163.5
11	6‐8‐2023	22.1	5.3	1	1	U	76.4	56.0

*Note*: “Duration” reports the total time device was attached to the turtle and “Video duration” reports total length of video recorded.

Abbreviations: SCL, Straight Carapace Length; U, unknown sex.

## 3 RESULTS AND DISCUSSION

**FIGURE 2:**
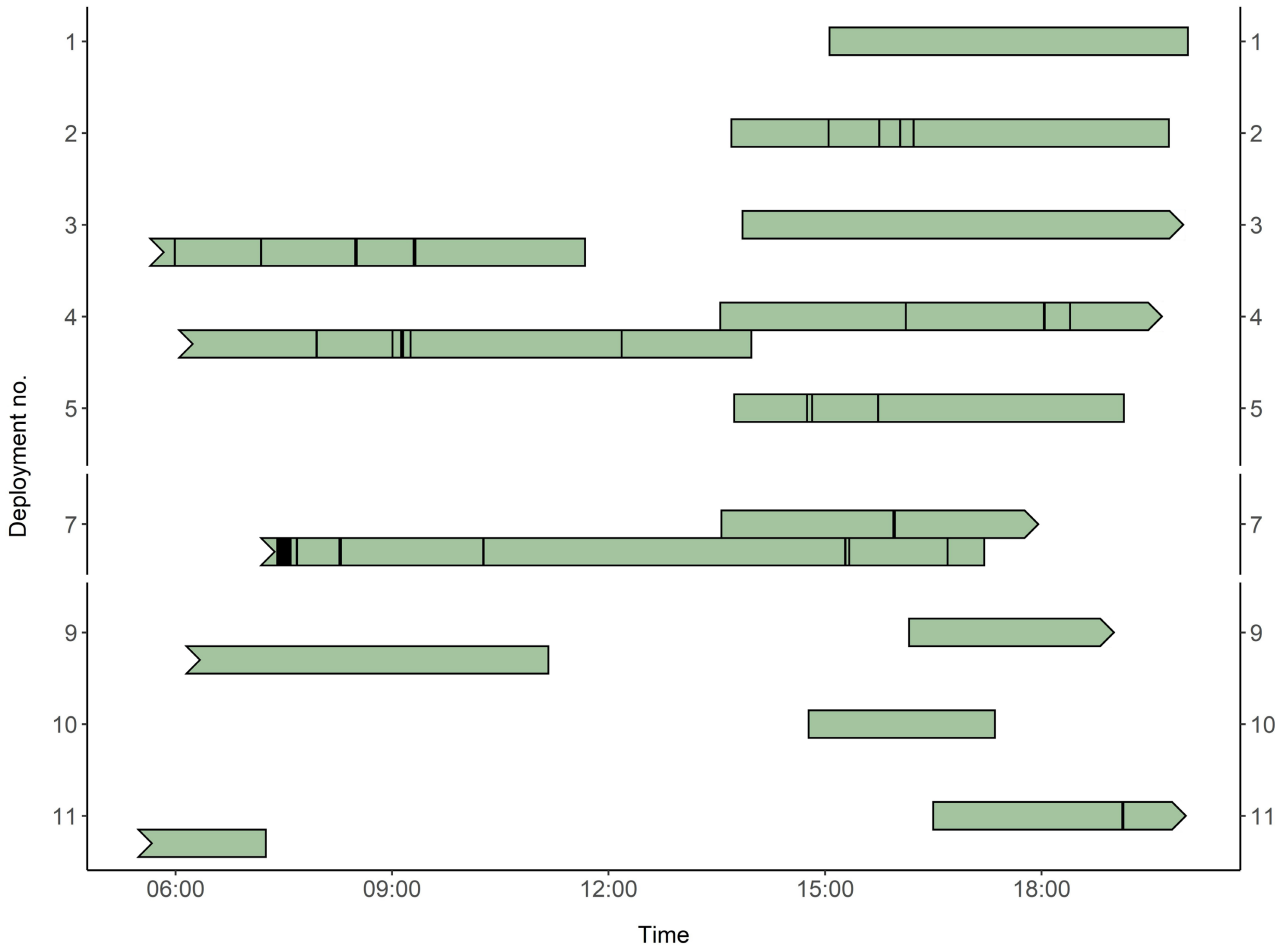
Deployment duration and timing for successful deployments. Green turtle intraspecific interactions observed with an animal‐borne camera. Bars represent the duration of video for each deployment, with stacked bars used to represent deployments spanning multiple days (deployments 6 and 8 not shown due to limited video; Table 1). For multiple‐day deployments, arrows mark the end of day one, and indents mark the beginning of day two. Vertical lines mark intraspecific interactions, with vertical line width representing interaction duration. Video collection was restricted to daylight hours. Thirty‐two interactions were observed over 73 h of footage.

**FIGURE 3:**
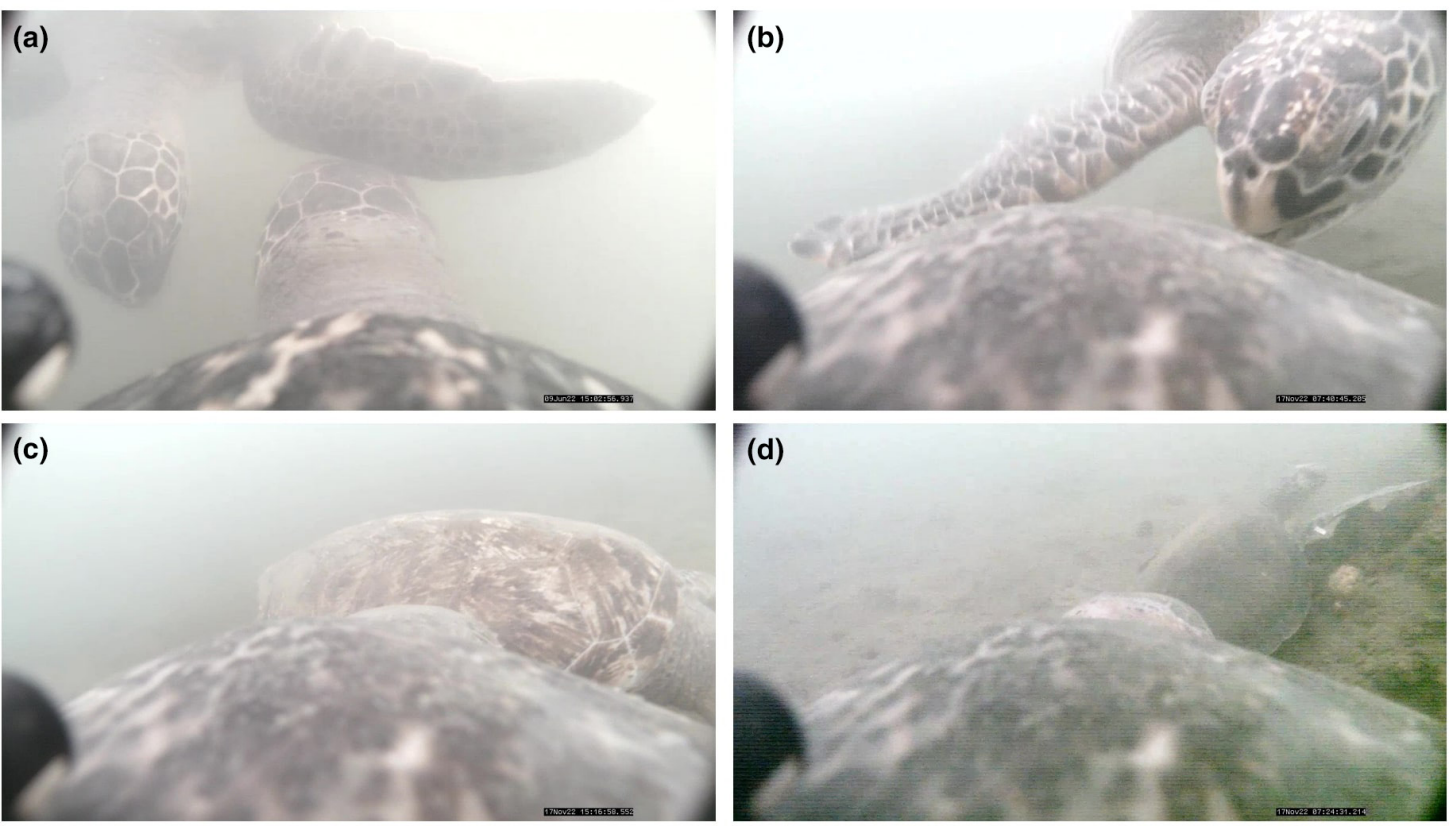
Instances of intraspecies interaction between green turtles in San Diego Bay, captured by an animal‐borne camera.

The camera system was deployed on 11 turtles during the study period, yielding a total of 73 h of video (Table 1). Due to photic limitations, all video was recorded during the day (i.e., between sunrise and sunset). We sampled seven putative females, one male, and three turtles of unknown sex, ranging in straight carapace length from 70.0 to 103.2 cm and in body weight from 46.5 to 163.5 kg (Table 1). Deployment durations ranged from 1.0 to 30.8 h. On two occasions the camera system released prematurely and recorded 1 h or less of usable video; these deployments were excluded from video analyses. For the 9 viable deployments, water visibility in the video was estimated to be <3 m, providing limited information beyond the immediate field of view of the turtle. Video revealed 32 instances of intraspecific interactions, at an average rate of 0.4 ± SD 0.3 interactions per hour over the 10 deployments. Turtle‐turtle interactions took place in the water column and on the seafloor, ranging in duration from 0.07 to 16.67 min (Figure 2), with the longest interaction being two turtles resting in close proximity on the bay floor. Longer interactions primarily occurred while turtles were on the seafloor, and usually were characterized by turtles resting near one another (Figure 3). Linear regressions did not provide any evidence for size‐based differences in social interaction behavior; SCL was not a significant predictor of interaction rate (*p* = .86) or proportion of video time spent interacting (*p* = .13), though we note that inferences here were limited by sample size and constrained by a relatively narrow range of body sizes sampled (70–103.2‐cm SCL). Although multiple fish species were observed in the video frame, only one instance of interspecific interaction was observed, a brief encounter with a California spiny lobster (*Panulirus interruptus*).

**FIGURE 4:**
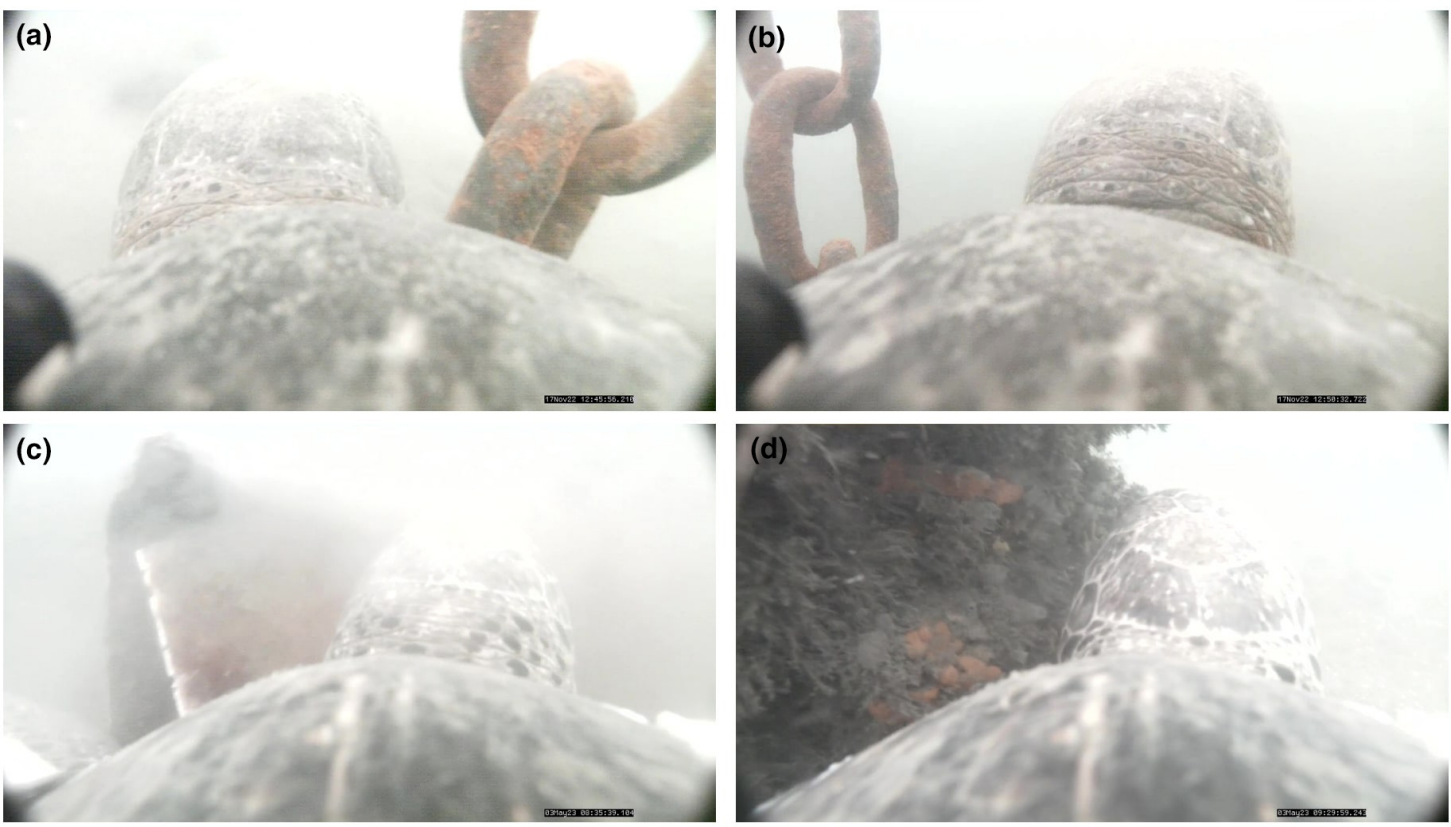
Evidence of green turtles in San Diego Bay exhibiting rubbing behavior on a buoy chain (a, b), and sunken metal debris (c, d), captured by an animal‐borne camera.

Turtle‐borne video also revealed novel interactions between green turtles and anthropogenic structures in the urban environment of SDB. We observed four turtles rubbing against man‐made benthic structures a total of seven times. In these instances, six unique hard structures were used. For example, one turtle scratched on a buoy chain for 38.33 min, left and returned to the same buoy chain 13.0 min later to spend another 32.4 min rubbing (Figure 4a,b). These were counted as separate instances. Of the five other structures used, one was an apparent sunken vessel and the other four were unidentified metal structures on the seafloor, two of which had substantial biofouling (Figure 4). We observed turtles rubbing their neck, shoulders, and flippers against these structures. Notably, most observed rubbing behavior was of skin, as opposed to carapace. On two occasions, we observed turtles congregating at benthic structures for apparent communal resting. No interactions with non‐anthropogenic structures were observed.

Given the number of turtles living in SDB (Eguchi et al., 2010, NOAA Unpubl. data), it follows that they would be observed near one another. The sharing of a relatively small space does not on its own suggest sociality. What would reveal more about their sociality is evidence of turtles actively seeking out other turtles, though this “active” aspect of their behavior is challenging to assess. Based on our video, we categorized interactions as either active or coincidental based on clear changes in behavior or swimming direction upon the sight of another turtle. This included turtles in the water column changing direction to follow another turtle passing by, as well as turtles approaching other resting or swimming turtles. We acknowledge that in cases when at‐large turtles approached the camera‐equipped turtle, the camera may have had an influence on said behavior. Despite this caveat, instances where other turtles approached the camera‐equipped turtle were counted as active interactions, given that they likely still suggest interest or curiosity in other individuals. In addition, because our device was forward‐facing, interactions or behaviors that involved the out‐of‐frame parts of the turtle would have gone largely unobserved.

Of 32 observed interactions, we categorized 18 as active. None of the active interactions reflected agonistic behavior, suggesting that despite high rates of socialization, intraspecies competition for resources and space may not be challenges faced locally. This contrasts with evidence from other contexts in which turtles have been documented exhibiting agonistic behavior towards other turtles (biting and chasing) to gain access to food and resting spots (Lamont et al., 2023 [loggerhead, green, and Kemp's ridley sea turtles], Schofield et al., 2022 [loggerhead sea turtles], Smolowitz et al., 2015 [loggerhead sea turtles]). The lack of apparent competition suggests that SDB may be below carrying capacity for green turtles. We note that many deployments took place during the regional green turtle nesting season (Delgado‐Trejo & Alvarado‐Diaz, 2012), which would temporarily reduce the number of turtles in the bay, although the majority of the population is immature (NOAA unpubl. data). Although we did not account for a variety of factors that likely influence turtle behavior (e.g., environmental), the variety in response when seeing another turtle (approaching, ignoring, following) raises questions about social preferences, that is, whether turtles recognize and develop preferential familiarity with other individuals.

A subset of interactions (*n* = 4) entailed group resting at sunken, man‐made structures, and we posit that these structures could act solely as a common landmark or may provide some additional benefit to the turtles. We frequently observed turtles rubbing on these structures, though we did observe resting at benthic structures where no rubbing was observed. Green, loggerhead, and Kemp's ridley sea turtles have all been observed exhibiting similar rubbing and resting behaviors at anthropogenic structures in the northern Gulf of Mexico (Siegfried et al., 2022). Other potential benefits include shelter; Thomson and Heithaus (2014) found that green turtle congregations were more likely to occur near benthic hard structures and attributed this to protection from predators. Years of research in SDB suggest that the habitat largely lacks green turtle predators, indicating that these structures likely serve another purpose locally, although it is possible that evolved behavioral tendencies drive an attraction to hard structures for safety and shelter despite no predation risk. Yet, several other factors could be at play. For instance, in SDB's primarily sand‐ and mud‐based environments, benthic structures provide surfaces for settlement by potential prey species such as sponges (Porifera, Figure 4d). Although not directly observed, we conjecture that turtles may seek out these structures for possible foraging opportunities.

Rubbing behavior in SDB mirrors previous findings for green turtles in non‐urban settings where rubbing was observed on naturally occurring surfaces (Heithaus et al., 2002; Thomson & Heithaus, 2014). Heithaus et al. (2002) hypothesized that the behavior serves a cleaning purpose (removing of epibiota) in ecosystems where turtles do not obtain that service from symbionts. This hypothesis aligns with our findings, as no symbiotic relationship involving turtles has been observed in SDB. Additionally, shell biofouling in green turtles has been documented year‐round at the nearby La Jolla Shores foraging area (30 km NNW from our SDB study site; Hanna et al., 2021) and anecdotal observations suggest that some SDB green turtles face the same challenge, though not to the same level. Additionally, there may be other benefits from this behavior, such as exfoliation. On two occasions, turtles partially detached the device while rubbing against structures. The regular use of metal structures by multiple green turtles in SDB suggests that these hard surfaces provide a consistent function and, although the ultimate possible benefits to turtles merit further study, these behaviors showcase local adaptation to urban environments.

Our study is one of the first to employ cutting‐edge pop‐off cameras that were highly customized for deployment on sea turtle carapaces (Hounslow et al., 2023; Jeantet et al., 2020). At the early stages of this exciting new technology, we point to some clear benefits that the CATS pop‐off camera has over many more common methods. First, precise GPS locations provided by an Iridium‐enabled locator beacon help to greatly simplify retrieval (when equipped). Second, a suite of high‐quality sensors provides incredible data volumes on fine‐scale movement, a subject of future research in SDB. Third, detachment with nothing left on the turtle is an improvement on methods using, for example, epoxy. Finally, the device is entirely wireless, including data transfer over local wifi, minimizing the risk of water infiltration. We look forward to seeing how this technology continues to develop and improve our understanding of the behavior of otherwise cryptic marine megafauna.

In sum, the high frequency of interactions exhibited relatively consistently across multiple individuals provides local evidence for sociality in this population of green turtles. In addition, the repeated use of, and congregation at unnatural structures provides information on the less‐understood aspects of industrial habitat use, both locally and more broadly. Technological advancements have enabled us to provide new insight into the life history of an extensively studied green turtle population, and our findings reinforce that we still have much to learn about these cryptic reptiles. Our work offers a framework that can inform future fine‐scale analyses of sea turtle behavior. Subsequent local research could leverage the frequency of interactions to study social interactions in more detail, including hypothetical communication methods. Longer deployments, night‐vision cameras, and hydrophones could all serve to expand upon our knowledge of these green turtles and their social habits.

## AUTHOR CONTRIBUTIONS

**Cameron M. Mullaney:** Conceptualization (supporting); data curation (supporting); formal analysis (equal); investigation (equal); writing – original draft (lead); writing – review and editing (equal). **Jeffrey A. Seminoff:** Conceptualization (equal); funding acquisition (lead); methodology (equal); project administration (lead); resources (lead); supervision (supporting); writing – review and editing (equal). **Garrett E. Lemons:** Conceptualization (supporting); investigation (equal); methodology (equal); project administration (equal); resources (equal); writing – review and editing (equal). **Bryant Chesney:** Conceptualization (supporting); funding acquisition (lead); resources (equal); writing – review and editing (equal). **Andrew S. Maurer:** Conceptualization (equal); data curation (equal); formal analysis (equal); investigation (lead); methodology (equal); software (equal); supervision (lead); writing – review and editing (equal).

## Data Availability

Video data used for this project are available via the following link: https://doi.org/10.5061/dryad.05qfttf9t.
